# Saliency Detection with Bilateral Absorbing Markov Chain Guided by Depth Information

**DOI:** 10.3390/s21030838

**Published:** 2021-01-27

**Authors:** Jiajia Wu, Guangliang Han, Peixun Liu, Hang Yang, Huiyuan Luo, Qingqing Li

**Affiliations:** 1 Changchun Institute of Optics, Fine Mechanics and Physics, Chinese Academy of Sciences, Changchun 130033, China; wujiajia17@mails.ucas.ac.cn (J.W.); liupx@ciomp.ac.cn (P.L.); yanghang@ciomp.ac.cn (H.Y.); luohuiyuan@ciomp.ac.cn (H.L.); liqingqing17@mails.ucas.ac.cn (Q.L.); 2 School of Optoelectronics, University of Chinese Academy of Sciences, Beijing 100049, China

**Keywords:** saliency detection, absorbing Markov chain, depth information, cross-modal multi-graph learning

## Abstract

The effectiveness of depth information in saliency detection has been fully proved. However, it is still worth exploring how to utilize the depth information more efficiently. Erroneous depth information may cause detection failure, while non-salient objects may be closer to the camera which also leads to erroneously emphasis on non-salient regions. Moreover, most of the existing RGB-D saliency detection models have poor robustness when the salient object touches the image boundaries. To mitigate these problems, we propose a multi-stage saliency detection model with the bilateral absorbing Markov chain guided by depth information. The proposed model progressively extracts the saliency cues with three level (low-, mid-, and high-level) stages. First, we generate low-level saliency cues by explicitly combining color and depth information. Then, we design a bilateral absorbing Markov chain to calculate mid-level saliency maps. In mid-level, to suppress boundary touch problem, we present the background seed screening mechanism (BSSM) for improving the construction of the two-layer sparse graph and better selecting background-based absorbing nodes. Furthermore, the cross-modal multi-graph learning model (CMLM) is designed to fully explore the intrinsic complementary relationship between color and depth information. Finally, to obtain a more highlighted and homogeneous saliency map in high-level, we structure a depth-guided optimization module which combines cellular automata and suppression-enhancement function pair. This optimization module refines the saliency map in color space and depth space, respectively. Comprehensive experiments on three challenging benchmark datasets demonstrate the effectiveness of our proposed method both qualitatively and quantitatively.

## 1. Introduction

The salient object detection (SOD) is a fundamental task in computer vision, which attempts to imitate the human visual attention mechanism to locate and segment the interesting or attractive regions in a scene. It has been widely applied to a variety of vision tasks, such as image segmentation [1], resizing [2], enhancement [3], quality assessment [4], recognition [5], and matching [6]. In fact, the human visual system can not only intuitively capture the appearance of objects, but also perceive the depth information from the scene. Benefiting from the development of 3D sensing technology, the depth information can be captured more conveniently and accurately. Therefore, the RGB-D saliency detection using depth information is attracting more and more attention. Moreover, the effectiveness of depth information has been fully proved in other computer vision tasks, such as motion segmentation [7] and people re-identification [8].

Given a pair of RGB-D (RGB + depth) images, the task of the RGB-D saliency detection aims to predict a saliency map and extract the salient regions by exploring the complementary information between color image and depth data. Furthermore, existing RGB-D saliency detection models mainly use depth information in two ways. One is based on depth features [9,10,11,12,13,14,15,16,17,18,19,20,21,22,23,24,25], which focuses on taking depth information as an explicit supplementary feature of color features. In [12], Cheng et al. calculate the saliency map with additional depth information through color contrast, depth contrast, and spatial bias extended from 2D to 3D, which also proves that depth information is beneficial to visual saliency analysis in complex scenes. In order to fully explore the potential color and depth cues in the whole saliency processing process, Peng et al. [16] propose an evolution strategy to introduce depth information into super-pixel generation, initial saliency map generation, and saliency propagation. In [24], Fang et al. propose a united stereoscopic saliency model, which combines depth-guided background prior, boundary background, and compactness based on disparity to estimate the initial saliency map. The map is refined by using the spatial dissimilarity features under reduced dimensions and central preference. Zhu et al. [17,18] directly use the depth map to generate the depth feature saliency and merge it with the color features saliency, then optimize the saliency map by combining the center dark channel prior (CDCP) or background elimination model. In [21], Song et al. generate different saliency measures based on multi-level features at different scales and perform discriminative saliency fusion through a random forest regressor to obtain the final saliency result. Aiming at the problem that the robustness of the saliency detection algorithm is not satisfied in some complex situations containing multiple objects or complex background, Zhu et al. [20] propose a multilayer backpropagation algorithm based on depth mining, which extracts depth cues from four different saliency layers to improve performance.

The other is based on depth measurement [26,27,28,29,30,31,32,33,34,35,36], which aims to obtain implicit attributes such as shape and contour from the depth map by designing depth measurement algorithms. Ren et al. [27] propose the normalized depth prior and the global-context surface orientation prior. These prior can highlight near objects, weaken distant objects and reduce the saliency of severely inclined surfaces (such as the ground plane or ceilings). In [26], instead of using absolute depth, Ju et al. propose an anisotropic center-surround difference (ACSD) measure that considers the global depth structure to calculate and perceive the depth saliency map. Since the background usually contains the regions with a large change in depth compared to the neighborhood, this leads to a higher contrast in this region. In response to this problem, Feng et al. [28] design a local background enclosure (LBE) feature to capture the spread of angular directions, which quantifies the proportion of the object boundary that is in front of the background from the depth map. In [33], Wang et al. propose a multi-stage salient object detection framework based on minimum barrier distance transformation and multi-layer cellular automata (MCA). The framework integrates multiple visual features and priors including background prior, 3-D spatial prior and depth bias. In general, the depth-feature based method is an intuitive and simple to achieve the RGB-D saliency detection, which ignores the potential attributes in the depth map. By contrast, the depth-measurement based method aims to refine the saliency results by using implicit information.

However, limited by the technology of the depth sensor, not all depth information is accurate and practicable. In another word, when the depth maps are accurate, they can provide precise depth information to facilitate saliency detection, on the contrary, they may cause detection failure when the depth maps are poor. In order to handle this problem, Cong et al. [37] present a depth confidence measure to assess the reliability of the depth map and control the fusion ratio of depth features and color features in the saliency model. In addition, in [38], a novel saliency detection model is proposed that combines the implicit and explicit features of the depth map, its main idea is to transfer the existing RGB saliency detection model to RGB-D images with the help of depth constraint, so that it can inherit the saliency performance of RGB image. To a certain extent, the utilization efficiency of depth information is improved, but it also has a problem that the algorithm greatly relies on the performance of the RGB saliency detection algorithm. Therefore, how to effectively fuse depth information to enhance the detection of salient objects is still challenging. Moreover, the detection results of the above algorithms are mostly not ideal for scenes where the object touches the boundary.

To tackle these problems, we propose a saliency detection model with the bilateral absorbing Markov chain guided by depth information. The model includes three progressive processing stages. At the first stage, we explicitly combine depth features with color features to calculate the low-level saliency information based on background prior and contrast prior. In the second stage, we design a bilateral absorbing Markov chain model based on the background seed selection mechanism and cross-modal multi-graph learning model. In this stage, we can obtain mid-level foreground-based and background-based saliency maps by using low-level saliency cues of first stage. In the final stage, to further improve the performance of our algorithm, we propose a depth-guided optimization module to obtain a more homogeneous salient region.

The main contributions of our paper can be summarized as:

1. A multi-stage RGB-D saliency detection framework with the bilateral absorbing Markov chain model is proposed. The framework can make full use of the explicit and implicit information in the depth map and explore the complementary relationship between the modes.
2. The background seed screening mechanism is designed to solve the boundary touch problem. Moreover, the cross-modal multi-graph learning model is designed for implicitly fusing color and depth information by the learning.
3. To preferably highlight the salient regions, we design a depth-guided optimization module which combines cellular automata and suppression-enhancement function pair.

## 2. Methodology

**Figure 1:**
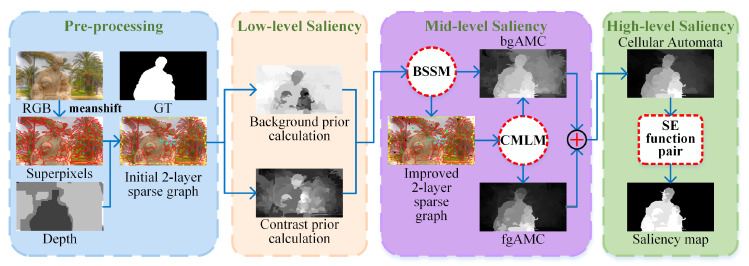
Flowchart of the proposed method. BSSM: background seed screening mechanism; CMLM: cross-modal multi-graph learning model; bgAMC and fgAMC denote background-based and foreground-based saliency maps based on absorbing Markov chain respectively; SE function pair represents suppression-enhancement function pair.

This section describes the proposed method in detail, and the overall framework is shown in Figure 1. The algorithm mainly consists of four subsections: pre-processing, low-level saliency cues calculation, mid-level saliency maps generation and high-level saliency optimization.

### 2.1. Initial Two-Layer Sparse Graph Constrution

Given an RGB image and an aligned depth map, we first convert the RGB image to the CIELAB color space and segment it into *N* superpixels using mean shift [39] algorithm. The superpixel is a small region in the image composed of a series of adjacent pixels with similar features e.g., color, brightness, texture, etc. Then, we construct an initial two-layer sparse graph G=(V,E) such as [40], where $V=\{v_{i}|1\leq i\leq N\}$ denotes the nodes and $E=\{e_{ij}|1\leq i,j\leq N\}$ denotes the edges between nodes. The graph is generated by connecting each node to neighboring nodes and the most similar node sharing a common boundary with its neighboring nodes. It is worth to notice that the nodes on the four boundaries of the image are connected to each other to reduce the geodesic between the background nodes. As [40] proves, compared with the ordinary two-layer graph, the two-layer spares graph can effectively avoid the interference from surrounding redundant nodes.

In this work, we utilize the pre-trained FCN-32s network [41] to extract the color feature vector, the Euclidean distance $c_{ij}$ in RGB color space and depth difference $d_{ij}$ between superpixels i and j are defined as

(1) $$c_{ij}=∥x_{i}-x_{j}∥$$

and

(2) $$d_{ij}=|d_{i}-d_{j}|$$

where $x_{i}$ is the mean color feature vector of superpixel i, and $d_{i}$ denotes the mean depth value of superpixel i. The similarity $a_{i}{}_{j}$ between superpixels i and j is defined as

(3) $$a_{i}{}_{j}=a_{ij}^{c}\cdot {\left(a_{ij}^{d}\right)}^{\varepsilon }$$

where the coefficient ε adjusts the weight of depth information and set as 0.5, $a_{ij}^{c}$ and $a_{ij}^{d}$ represent the color similarity and depth similarity respectively, and are defined as

(4) $$a_{ij}^{c}=e^{-\frac{c_{ij}}{\sigma^{2}}}$$

and

(5) $$a_{ij}^{d}=e^{-\frac{d_{ij}}{\sigma^{2}}}$$

where $\sigma^{2}$ is a parameter to control strength of the similarity which is set to 0.1. The affinity matrix $W={\left[w_{ij}\right]}_{N\times N}$ of the graph is defined as the similarity between two superpixels,

(6) $$w_{ij}=\{\begin{array}{ll} a_{ij}, & \mathrm{if}j\in \Omega_{i}\\ 0, & \mathrm{otherwise} \end{array}$$

where $\Omega_{i}$ is the neighbors of superpixel i based on the initial two-layer sparse graph.

### 2.2. Low-Level Saliency Cues Calculation Using Color and Depth Cues

In this part, explicitly combining color and depth cues, we calculate low-level saliency information based on background prior and contrast prior. The saliency prior maps are shown in Figure 1.

#### 2.2.1. Background Prior Calculation

We adopt boundary connectivity [42] to generate the background prior map, which is defined as

(7) $$S_{bp}(i)=1-\exp(-\frac{BndCon^{2}(i)}{2\sigma_{bndCon}^{2}})$$

in which BndCon(i) refers to the value of boundary connectivity for superpixel i and $\sigma_{bndCon}$ is a weighting factor for boundary connectivity. Here empirically sets $\sigma_{bndCon}^{2}=1$. This background measure is robust to the normal cases and can effectively eliminate most background regions.

#### 2.2.2. Region Contrast Prior Calculation

Human attention tends to focus on those image regions that contrast strongly with the surroundings. Therefore, we calculate a region contrast similar with [43], which integrates depth features and rich color features together. Then, compared to all other regions, we compute its saliency value by measuring its depth and color combined contrast,

(8) $$S_{rc}(i)=\sum_{j=1,j\neq i}^{N}a_{ij}D_{o}\left(i,j\right)Area\left(j\right)$$

where $D_{o}\left(i,j\right)$ represents the Euclidean spatial distance between the superpixel i and j, $Area\left(v_{j}\right)$ is the area ratio of superpixel j compared with the whole image.

### 2.3. Mid-Level Saliency Maps Generation by Bilateral Absorbing Markov Chain

Inspired by [44], we design a bilateral absorbing Markov chain model, which combines multi-layer color features and depth features to obtain learned transition probability matrixes, and generate mid-level saliency maps. Most of the saliency models have poor detection results when the salient object is not in the center of the image, especially in the case of some salient regions touch the image boundary. To handle this situation in ours model, we propose a background seed screening mechanism (BSSM) to improve the graph model and better select background-based absorbing nodes. Moreover, we present a cross-modal multi-graph learning model (CMLM) to obtain the learned affinity and transition probability matrixes, which can make full use of the complementarity of color and depth information.

#### 2.3.1. Absorbing Markov Chain for Saliency Detection

To facilitate the understanding, we give a brief introduction to the principle of absorbing Markov chain [45,46]. For a given set of states $S=\left\{s_{1},s_{2},\ldots ,s_{k}\right\}$, the probability of moving from state $s_{i}$ to the next state $s_{j}$ is expressed as the transition probability $p_{ij}$, which does not depend on the chain before the current state. An absorbing Markov chain contains at least one absorbing state ($p_{ii}=1$), and starts from every transient state, a certain absorbing state can be reached. For an absorbing chain with r absorbing states and t transient states, the canonical form of the transition matrix P is as follows,

(9) $$P\to \left(\begin{array}{cc} Q & R\\ 0 & I \end{array}\right)$$

where $Q\in {\left[0,1\right]}^{t\times t}$ represents the transition probability of any pair of t transient states, while $R\in {\left[0,1\right]}^{t\times r}$ represents the transition probability between any transient state and absorbing state. 0 is the r×t zero matrix and I is the r×r identity matrix. Furthermore, the fundamental matrix N is computed [45],

(10) $$N={\left(I-Q\right)}^{-1}=I+Q+Q^{2}+\cdot \cdot \cdot$$

where $n_{ij}$ of N can describe the expected number of times from transient state $s_{i}$ to transient state $s_{j}$ in the absorbing chain.

Then the absorption probability for each transient state to reach any absorbing state can be defined as [46],

(11) B=NR

where $b_{ij}$ of B indicates the absorption probability from transient state $s_{i}$ to transient state $s_{j}$.

Traditional saliency detection models based on absorbing Markov chain generally mirror image boundary superpixels as absorbing nodes (or states), and all others as transient nodes. Then, the transition matrix P is constructed according to the similarity (the transition probability) between nodes. The saliency value is measured by the absorption probability, the higher the absorption probability of the node, the more similar to the absorbing nodes.

#### 2.3.2. Background Seed Screening Mechanism

**Figure 2:**
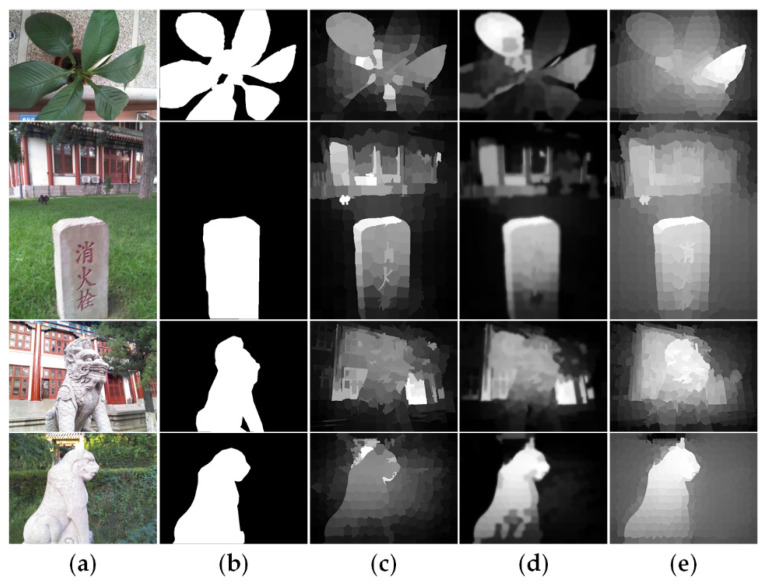
Traditional saliency detection models based on absorbing Markov chain. (**a**) RGB images. (**b**) Ground truth. (**c**) Saliency maps generated by [45]. (**d**) Saliency maps generated by [46]. (**e**) Saliency maps generated by [44].

Generally, traditional saliency detection models based on absorbing Markov chain [44,45,46] usually mirror image edge superpixels as absorbing nodes and simply connect all edge superpixels in pairs. However, as shown in Figure 2, when the salient object touches the image boundary, the mirroring will mistakenly regard the foreground nodes as background-based absorbing nodes, thus suppressing the saliency of the foreground regions or causing detection failure. Similarly, if the edge nodes contain foreground nodes, the full connections between them may be poorly robust. To overcome them, we propose a background seed screening mechanism (BSSM) for improving the two-layer sparse graph and selecting better background-based absorbing nodes. This mechanism removes the nodes that may belong to the foreground from the edge nodes. Furthermore, in order to increase the diversity of the background and restrain the background regions, a small number of random non-edge background nodes are selected to form a new edge node set and a background-based absorbing node set. Moreover, to obtain more homogeneous salient regions, we design the non-local connection similar to [47]. Next, we will introduce the construction process of the background seed screening mechanism and the non-local connections in detail.

**Figure 3:**
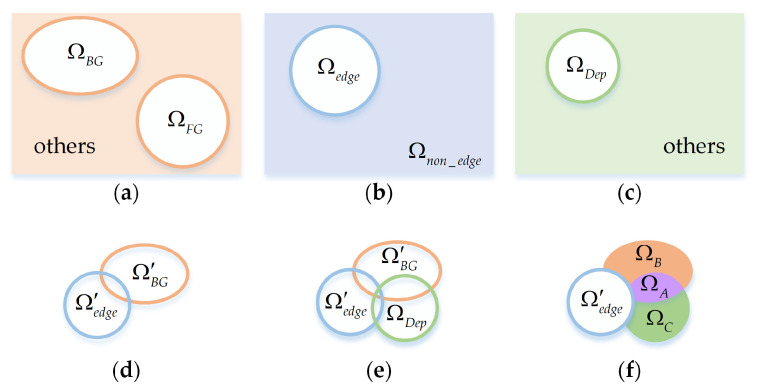
The process diagram of the background seed screening. (**a**) The node sets based on saliency attribute; (**b**) The node sets based on location attribute; (**c**) The node sets based on depth attribute; (**d**) The node sets after removing the foreground nodes; (**e**) The diagram of the comprehensive relationship between the three attributes; (**f**) The final node sets after classifying.

To facilitate understanding, we provide a schematic diagram in Figure 3, which describes the main screening process of the background seeds. First, according to the attributes of saliency, position and depth, all nodes are classified as three categories. As shown in Figure 3a, based on the low-level background prior $S_{bp}$, we divide all nodes into background seed set $\Omega_{BG}$, foreground seed set $\Omega_{FG}$ and others.

(12) $$\Omega_{BG}=\left\{i|S_{bp}(i)>0.9\right\}$$

(13) $$\Omega_{FG}=\left\{i|S_{fp}(i)>th_{FG}\right\}$$

where $S_{fp}$ represents the foreground prior,

(14) $$S_{fp}(i)=1-S_{bp}(i)$$

(15) $$th_{FG}>\frac{3\cdot mean(S_{fp})+\max(S_{fp})}{4}$$

According to the position attribute, the nodes can be classified as edge node set $\Omega_{edge}=\left\{i|i\in \mathrm{edge}\right\}$ and non-edge node set $\Omega_{non\_edge}=\left\{i|i\notin \mathrm{edge}\right\}$ as shown in Figure 3b.

Considering that objects far away from the camera are likely to belong to the background, as shown in Figure 3c, we use the depth threshold to divide the nodes into depth-based background seed set $\Omega_{Dep}$ and others.

(16) $$\Omega_{Dep}=\left\{i|d_{i}>1.2*th_{Dep}\;\mathrm{and}\;i\notin \Omega_{FG}\right\}$$

(17) $$th_{\mathrm{Dep}}>\frac{3*mean(d_{i})+\max(d_{i})}{4},i\in \Omega_{BG}$$

To alleviate the boundary touch problem and select background seeds more accurately, we utilize k-means algorithm to filter out the foreground nodes in the background seed set $\Omega_{BG}$ and edge node set $\Omega_{edge}$. More specifically, we cluster the sets of $\Omega_{BG}$, $\Omega_{edge}$ and $\Omega_{FG}$ to find the nodes that are similar with the foreground seeds $\Omega_{FG}$. Figure 3d is the filtered result: new edge node set ${\Omega^{\prime }}_{edge}$ and background seed set ${\Omega^{\prime }}_{BG}$. In Figure 3e, we take depth information into consideration in the process of background seed screening. In Figure 3f, non-edge background seeds and depth-based background seeds are further divided into three sub-sets: $\Omega_{A}$, $\Omega_{B}$, and $\Omega_{C}$. It is obvious that the seeds in $\Omega_{A}$ satisfy both background probability and depth with high values, while the seeds in $\Omega_{B}$ and $\Omega_{C}$ only satisfy the requirement of high background probability or high depth value, respectively.

**Figure 4:**
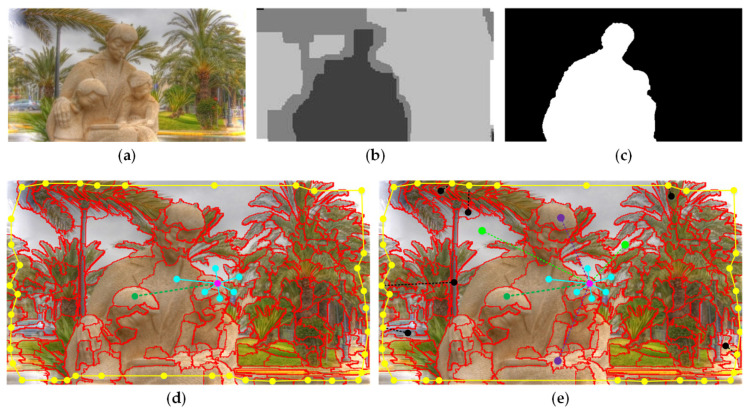
The construction and comparison of the proposed graph model. (**a**) Input RGB image. (**b**) Input depth image. (**c**) Ground truth. (**d**) A diagram of the connections of one of the nodes based on initial two-layer sparse graph. A node (illustrated by a pink dot) connects to its adjacent nodes (blue dots and connections) and the most similar node (dark green dots and connections) sharing common boundaries with its adjacent nodes. All edge nodes are connected to pairs (yellow dots and local connections). (**e**) A diagram of the connections of one of the nodes based on improved two-layer sparse graph. Different from the initial graph, the new edge nodes first remove some foreground nodes which are in the image boundary (the nodes at the bottom edge of image), and further join a small number of non-edge background nodes (black nodes). Each pair of the new edge nodes connects to each other (yellow and black dots and connections). Additionally, each node connects to the background seeds (light green dots and connections) and the foreground seeds (purple dots and connections).

Then, for guaranteeing the diversity of the background and suppressing the background more effectively, we combine a small number of non-edge nodes with ${\Omega^{\prime }}_{edge}$ and further form the final edge nodes $\Omega_{f\_edge}$. These non-edge nodes are randomly composed of 50% $\Omega_{A}$, 10% $\Omega_{B}$, and 50% $\Omega_{C}$. In the initial two-layer sparse graph, to reduce the geodesic distances of nodes, all edge nodes are simply connected together. However, it may be poorly robust to the case when salient objects touch the image boundaries. Therefore, instead of the rough connections, we use the final edge nodes $\Omega_{f\_edge}$ connected in pairs to obtain a new two-layer sparse graph $G_{new}$. In addition, to obtain more consistent salient regions, we introduce the non-local connection into the graph. Specifically, it first sorts the foreground prior $S_{fp}$ and the region contrast prior $S_{rc}$ of all nodes, the top 50% of both are selected as foreground seeds, and the bottom 50% are selected as background seeds. For each superpixel, we connect it to two nodes that are randomly chosen from the two seed sets respectively. This connection mechanism is more conducive to highlight the foreground objects and suppress the background regions. The improved two-layer sparse graph with the non-local connection is visualized in Figure 4e.

**Figure 5:**
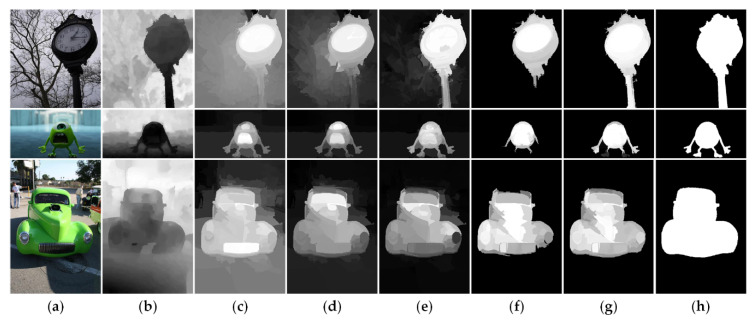
Visual comparisons of different graph-based results. (**a**) Original RGB images. (**b**) Original depth images. (**c**) Saliency maps produced by two-graph neighborhood graph. (**d**) Saliency maps produced by two-layer sparse graph. (**e**) Saliency maps produced by improved two-layer sparse graph based on background seed screening mechanism (BSSM). (**f**) Saliency maps produced by improved two-layer sparse graph without the non-local connections. (**g**) Saliency maps produced by improved two-layer sparse graph with the non-local connections. (**h**) Ground truth.

Moreover, Figure 5 demonstrate the effects of the proposed background seed screening mechanism (BSSM) and non-local connection. In Figure 5e, it is clearly observed that the background is well suppressed by the improved two-layer sparse graph based on background seed screening mechanism (BSSM). Figure 5g illustrates that the non-local connection can achieve more complete and consistent salient regions.

#### 2.3.3. Cross-Modal Multi-Graph Learning Model

The two-layer sparse graph constructs the connections among the local regions, which will restrict the range of random walk to the local regions. Therefore, the absorption time may be inaccurate, especially when the long-range smooth background distributes near the center of image. To overcome it, we have improved the graph model from the connection relationship in the above section. However, in the absorbing Markov chain model, another key influencing factor is the weight of the edges between nodes. Similar to Formula (3), most of the existing graph models directly weight depth and color cues to measure the similarity between nodes. However, the models do not consider the effect of color and depth information on saliency detection in different scenarios. For example, in some scenes, color is more reliable than depth, so a larger weight of color is needed. Conversely, if depth is more reliable, we need to strengthen the weight of depth. Therefore, we propose a cross-modal multi-graph learning model (CMLM), which fully explores the complementary relationship between color and depth in different scenarios. The learning model constructs a more accurate affinity matrix and captures the optimal fusion state of color and depth information.

Some algorithms [44,48] have constructed the affinity matrix by the learning. In [48], the learning model based on the single graph is proposed, which construct an approximate full affinity matrix by using the following equation,

(18) $$\underset{Y}{\min}\sum_{i,j=1}^{N}w_{ij}{\left\|y_{i}-y_{j}\right\|}^{2}+\mu \sum_{i=1}^{N}{\left\|y_{i}-i_{i}\right\|}^{2}$$

where $Y=[y_{i},y_{2},\ldots ,y_{n}]\in {\mathbb{R}}^{N\times N}$ is an affinity matrix optimized by unsupervised learning based on the original sparse affinity matrix. $y_{i}={\left[y_{i1},y_{i2},\ldots ,y_{iN}\right]}^{⊤}$ is a column vector indicating the degree of affinity between the node i and all other nodes, $i_{i}{}^{⊤}$ is the i-th column of an identity matrix Ι which indicates the similarity with itself. In Equation (18), the first item is a smoothing constraint item, which indicates the difference between $y_{i}$ and $y_{j}$. The two nodes are more similar, the value of first item will be lower. The second item is a self-restraint item, which emphasizes that no matter how we update the value of $y_{i}$ of node i, it should not be too different from its initial value. μ is a parameter that balances the relationship between the two items, μ>0.

Formula (18) is the learning process under the single-layer graph. To make full use of the complementarity of color and depth information, we explore feature spaces of multiple modes and develop a cross-modal multi-graph model to learn an affinity matrix. We use $\beta ={\left[\beta_{c}^{},\beta_{d}^{},\ldots \right]}^{⊤}$ to represent the set of multi-modal vectors, and its values indicate the importance of the corresponding affinity graph. In this work, we only adopt the modes of color and depth. $\beta_{c}^{}=[\beta_{c}^{\left(1\right)},\beta_{c}^{\left(2\right)},\ldots ,\beta_{c}^{\left(m\right)}]$ is a sub-vector of the color mode and m is the number of feature maps in color space. $\beta_{d}^{}=[\beta_{d}^{\left(1\right)},\beta_{d}^{\left(2\right)},\ldots ,\beta_{d}^{\left(n\right)}]$ is a sub-vector of the depth mode and n is the number of feature maps in depth space. $W_{c}^{\left(\nu \right)}={\left[w_{ij}^{c}{}^{\left(\nu \right)}\right]}_{N\times N}$ is the graph affinity matrix computed by the ν-th color feature and $W_{d}^{\left(\tau \right)}={\left[w_{ij}^{d}{}^{\left(\tau \right)}\right]}_{N\times N}$ is the graph affinity matrix computed by the τ-th depth feature. Then, the final learning affinity matrix optimization equation can be defined as

$$\underset{\beta ,Y}{\min}\sum_{\nu =1}^{m}{\left(\beta_{c}{}^{\left(\nu \right)}\right)}^{\gamma }\sum_{i,j=1}^{N}w_{ij}^{c}{}^{\left(\nu \right)}{\left\|y_{i}-y_{j}\right\|}^{2}+\sum_{\tau =1}^{n}{\left(\beta_{d}{}^{\left(\tau \right)}\right)}^{\gamma }\sum_{i,j=1}^{N}w_{ij}^{d}{}^{\left(\tau \right)}{\left\|y_{i}-y_{j}\right\|}^{2}+\mu \sum_{i=1}^{N}{\left\|y_{i}-i_{i}\right\|}^{2},$$

(19) $$s.t.\sum_{\nu =1}^{m}\beta_{c}{}^{\left(\nu \right)}+\sum_{\tau =1}^{n}\beta_{d}{}^{\left(\tau \right)}=1,0\leq \beta_{c}{}^{\left(\nu \right)},\beta_{d}{}^{\left(\tau \right)}\leq 1$$

where the parameter γ controls the weight distribution of all affinity matrices, ensuring that different-mode features can be fully utilized. Without this parameter, in some cases, it is possible that only partial features participate in the learning of affinity matrix, which may utilize the complementarity between different features insufficiently. The parameter μ and γ are set to 0.001 and 4 respectively. To facilitate the derivation, we rewrite the above objective function (19) in the form of matrix,

(20) $$J=\sum_{\nu =1}^{m}{\left(\beta_{c}^{\left(\nu \right)}\right)}^{\gamma }Tr\left(Y^{T}L_{c}^{\left(\nu \right)}Y\right)+\sum_{\tau =1}^{n}{\left(\beta_{d}^{\left(\tau \right)}\right)}^{\gamma }Tr\left(Y^{T}L_{d}^{\left(\tau \right)}Y\right)+\mu {\left\|Y-I\right\|}_{F}^{2}$$

where $L_{c}^{\left(\nu \right)}=D_{c}^{\left(\nu \right)}-W_{c}^{\left(\nu \right)}$ is the graph Laplacian matrix of the ν-th color feature, $D_{c}^{\left(\nu \right)}$ is the degree matrix and $d_{ii}^{c(\nu )}=\sum_{j=1}^{N}w_{ij}^{c(\nu )}$. Similarly, $L_{d}^{\left(\tau \right)}=D_{d}^{\left(\tau \right)}-W_{d}^{\left(\tau \right)}$ is the graph Laplacian matrix of the τ-th depth feature, $D_{d}^{\left(\tau \right)}$ is the degree matrix and $d_{ii}^{d(\nu )}=\sum_{j=1}^{N}w_{ij}^{d(\nu )}$. Tr(⋅) and $∥\cdot {∥}_{F}$ compute the trace and the Frobenius norm of the matrix separately. We can see that there are two unknown items β and Y to be solved in Equation (20), so we decompose it into two sub-problems to solve this optimization problem by iteration.

Fix β, Update Y:

(21) $$Y=\mu {\left(\sum_{\nu =1}^{m}\beta_{c}{}^{\left(\nu \right)}L_{c}^{\left(\nu \right)}+\sum_{\tau =1}^{n}\beta_{d}{}^{\left(\tau \right)}L_{d}^{\left(\tau \right)}+\mu I\right)}^{-1}$$

Fix Y, Update β:

(22) $$\beta_{c}{}^{\left(\nu \right)}=\frac{{\left(Tr\left(Y^{T}L_{c}^{\left(\nu \right)}Y\right)\right)}^{\frac{1}{1-\gamma }}}{\sum_{\nu^{\prime }=1}^{m}{\left(Tr\left(Y^{T}L_{c}^{\left(\nu^{\prime }\right)}Y\right)\right)}^{\frac{1}{1-\gamma }}+\sum_{\tau^{\prime }=1}^{n}{\left(Tr\left(Y^{T}L_{d}^{\left(\tau^{\prime }\right)}Y\right)\right)}^{\frac{1}{1-\gamma }}}$$

(23) $$\beta_{d}{}^{\left(\tau \right)}=\frac{{\left(Tr\left(Y^{T}L_{d}^{\left(\tau \right)}Y\right)\right)}^{\frac{1}{1-\gamma }}}{\sum_{\nu^{\prime }=1}^{m}{\left(Tr\left(Y^{T}L_{c}^{\left(\nu^{\prime }\right)}Y\right)\right)}^{\frac{1}{1-\gamma }}+\sum_{\tau^{\prime }=1}^{n}{\left(Tr\left(Y^{T}L_{d}^{\left(\tau^{\prime }\right)}Y\right)\right)}^{\frac{1}{1-\gamma }}}$$

To get the optimal solution of sub-problems, we utilize partial derivative and Lagrange Multiplier Method. The specific derivation process can refer to [48]. With the learned affinity matrix Y, we can calculate the transition matrixes of absorbing Markov chain. The final learned affinity matrix $W_{L}={\left[w_{ij}^{L}\right]}_{N\times N}$ is obtained by normalization,

(24) $$W_{L}=diag{\left(Y\right)}^{-1}\times Y$$

**Figure 6:**
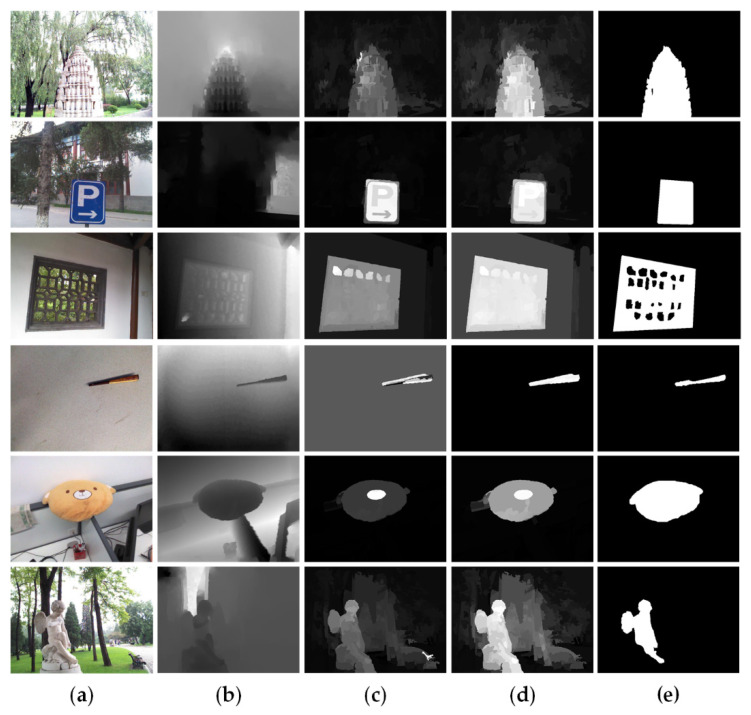
Visual comparisons of our proposed cross-mode multi-graph learning model (CMLM). (**a**) Original RGB images. (**b**) Original depth images. (**c**) Saliency maps based on single-mode multi-graph learning model (SMLM). (**d**) Saliency maps based on cross-modal multi-graph learning model (CMLM). (**e**) Ground truth.

Figure 6d shows the effects of the proposed cross-modal multi-graph learning model (CMLM). As it is illustrated, compared to single-mode multi-graph learning model (color mode), the proposed model is more precise to highlight the salient regions.

#### 2.3.4. Background-Based Saliency Map via Absorbing Markov Chain

**Figure 7:**
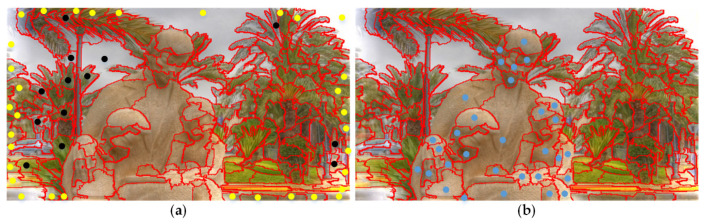
Relevant schematic diagrams of the bilateral absorbing Markov chain model. (**a**) The construction of background-based absorbing Markov chain model: mirror edge superpixels (yellow dots) and a few non-edge background superpixels (black dots) as virtual absorbing nodes; (**b**) The construction of foreground-based absorbing Markov chain model: mirror foreground superpixels (blue dots) as virtual absorbing nodes. Both set all superpixels on the entire image as transient nodes.

In this part, we select background-based absorbing nodes based on the above background seed screening mechanism. As is presented in Figure 7a, we mirror edge nodes ${\Omega^{\prime }}_{edge}$ and some non-edge background nodes as virtual absorbing nodes, and all nodes in the image as transient nodes. The non-edge background nodes are randomly composed of 50% $\Omega_{A}$, 50% $\Omega_{B}$ and 50% $\Omega_{C}$. The number of absorbing nodes is *r*. Then, the background-based affinity matrix $W_{L}^{}{}^{B}={\left[w_{ij}^{L}\right]}_{N\times r}$ can be obtained with Formula (24). Furthermore, the learned transition matrix is defined as

(25) $$P_{B}=\left[\begin{array}{cc} Q_{B}^{N\times N} & R_{B}^{N\times r}\\ 0_{}^{r\times N} & I_{B}^{r\times r} \end{array}\right]$$

where $Q_{B}^{N\times N}=D_{B}{}^{-1}W_{L}$, $R_{B}^{N\times r}=D_{B}{}^{-1}W_{L}^{}{}^{B}$, $D_{B}$ is the sum of the matrix $D_{1}$ and $D_{2}$, $D_{1}=diag\left\{d_{1}^{W_{L}},d_{2}^{W_{L}},\ldots ,d_{N}^{W_{L}}\right\}$ is the degree matrix of $W_{L}$, and $d_{i}^{W_{L}}=\sum_{i=1}^{N}w_{ij}^{L}$. $D_{2}=diag\left\{d_{1}^{W_{L}{}^{B}},d_{2}^{W_{L}{}^{B}},\ldots ,d_{N}^{W_{L}{}^{B}}\right\}$ is the degree matrix of $W_{L}^{}{}^{B}$, and $d_{i}^{W_{L}{}^{B}}=\sum_{i=1}^{r}w_{ij}^{L}$. According to Formula (11), we can calculate the absorption probability matrix $B_{B}=N_{B}R_{B}$, where $N_{B}^{}={\left(I-Q_{B}\right)}^{-1}$. Based on the above work, the saliency of the node i is defined as

(26) $$S_{bg}(i)=1-\sum_{j=1}^{r}b_{ij}$$

The background-based saliency map $S_{bg}$ is shown in Figure 1. Then, we mirror the nodes with the saliency value greater than the threshold th as the foreground-based absorbing nodes, which is illustrated in Figure 7b. The number of absorbing nodes is *k*.

(27) $$th=\left(mean\left(S_{bg}\right)+\max\left(S_{bg}\right)\right)/2$$

#### 2.3.5. Foreground-Based Saliency via Absorbing Markov Chain

Similarly, the foreground-based affinity matrix $W_{L}^{}{}^{F}={\left[w_{ij}^{L}\right]}_{N\times k}$ can be obtained with Formula (24), and the learned transition matrix is as follows,

(28) $$P_{F}=\left[\begin{array}{cc} Q_{F}^{N\times N} & R_{F}^{N\times k}\\ 0_{}^{k\times N} & I_{F}^{k\times k} \end{array}\right]$$

where $Q_{F}^{N\times N}=D_{F}{}^{-1}W_{L}$, $R_{F}^{N\times k}=D_{F}{}^{-1}W_{L}^{}{}^{F}$, $D_{F}$ is the sum of the matrix $D_{1}$ and $D_{2}{}^{\prime }$, $D_{2}{}^{\prime }=diag\left\{d_{1}^{W_{L}^{}{}^{F}},d_{2}^{W_{L}^{}{}^{F}},\ldots ,d_{N}^{W_{L}^{}{}^{F}}\right\}$ is the degree matrix of $W_{L}^{}{}^{F}$, $d_{i}^{W_{L}{}^{F}}=\sum_{i=1}^{k}w_{ij}^{L}$. According to Formula (11), the absorption probability matrix $B_{F}=N_{F}R_{F}$ is obtained, where $N_{F}^{}={\left(I-Q_{F}\right)}^{-1}$. In order to calculate the foreground-based saliency more accurately and eliminate the interference of weak correlated nodes, we sort each row of $B_{F}$ and select the top 60% of the nodes to calculate the final saliency value,

(29) $$S_{fg}(i)=\sum_{i=1}^{c}{b^{\prime }}_{ij}$$

where c=0.6∗k, and the foreground-based saliency map $S_{fg}$ is shown in Figure 1.

### 2.4. High-Level Saliency Map Optimization via Depth Guidance

In order to further highlight the salient regions and effectively explore the inner relationship between depth information and salient information, we design a depth-guided optimization module which combines cellular automata and suppression-enhancement function pair.

#### 2.4.1. Optimization via Cellular Automata

We perform a primary fusion of the saliency maps produced by the bilateral absorbing Markov chain model,

(30) $$S_{fb}(i)=0.4*S_{fg}(i)+0.6*S_{bg}(i)$$

Based on the improved two-layer sparse graph, we use the cellular automata [49] propagation mechanism to further optimize the fused saliency map. First, based on the learned affinity matrix $W_{L}$ and the color similarity matrix $A_{}^{c}={\left[a_{ij}^{c}\right]}_{N\times N}$, we construct an impact factor matrix $F={\left[f_{ij}\right]}_{N\times N}$,

(31) $$F=A_{}^{c}\cdot W_{L}$$

**Figure 8:**
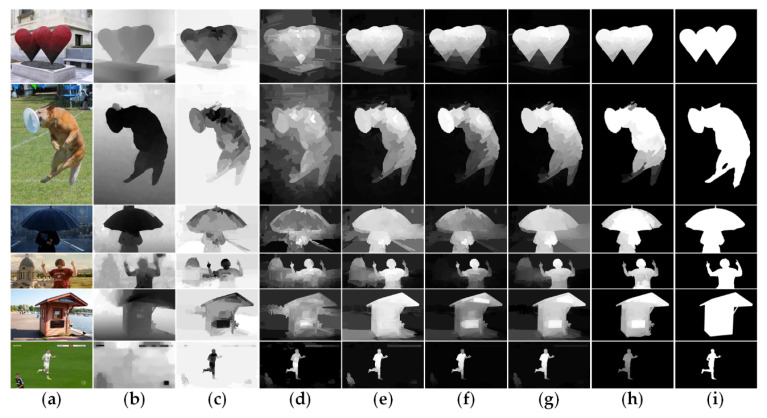
Visualization of the main saliency refinement process. (**a**) Original RGB images. (**b**) Original depth images. (**c**) Background prior probability maps combining color and depth cues. (**d**) Contrast prior probability maps combining color and depth cues. (**e**) Background-based saliency maps by absorbing Markov chain model. (**f**) Foreground-based saliency maps by absorbing Markov chain model. (**g**) Saliency maps optimized by cellular automata. (**h**) Saliency maps generate by the depth selective refinement mechanism based on suppression-enhancement function pair. (**i**) Ground truth.

Furthermore, all superpixel nodes (cells) are updated simultaneously through the following iteration rules,

(32) $$S^{h+1}=C^{*}\cdot S^{h}+(I-C^{*})\cdot F^{*}\cdot S^{h}$$

where I is the identity matrix. $F^{*}={\left[f_{ij}^{*}\right]}_{N\times N}$ and $C^{*}=diag\left\{c_{1}^{*},c_{2}^{*},\ldots ,c_{N}^{*}\right\}$ are normalized impact factor matrix and coherence matrix respectively,

(33) $$F^{*}=D_{f}^{-1}\cdot F$$

(34) $$c_{i}^{*}=a\cdot norm(1/\max(f_{ij}^{*}))+b$$

where $D_{f}^{}=diag\left\{d_{f1},d_{f1},\ldots ,d_{fN}\right\}$ is the degree of the matrix and $d_{fi}=\sum_{j}f_{ij}$. The constant coefficients a and b are set to 0.6 and 0.2, respectively, norm(⋅) means normalization function. Each cell can automatically evolve into a more accurate and stable state, and under the influence of the neighborhood, the salient regions are easier to be detected. The initial $S^{h}$ when h=0 is $S_{fb}$ in Equation (30), and the ultimate saliency map after h=10 time steps is denoted as $S_{CA}$, which is visualized in Figure 8g.

#### 2.4.2. Refinement via Depth Information

Cellular automata mainly explores the neighborhood relationship between the nodes in the color feature space, but ignores the spatial position information in the scene. Therefore, we use depth cues to enhance and refine the salient regions and suppress the background regions. In this work, we design a depth selective refinement mechanism by a suppression–enhancement function pair: the suppression function is used to suppress the background, and then an enhancement function is used to emphasize the salient regions through high-confidence depth seeds.

**Suppression function**: The regions far away from the camera have a higher probability of being the background and need to be suppressed. Therefore, we defined the suppression function as follows,

(35) $$S_{SF}(i)=\{\begin{array}{ll} S_{CA}(i), & \mathrm{if}\;S_{CA}(i)>0.7\mathrm{and}\;S_{d}(i)>0.5\mathrm{and}\;S_{CA}(i)>(th_{CA}+0.1)\\ S_{CA}(i)\cdot S_{d}(i), & \mathrm{if}\;S_{CA}(i)\leq th_{CA}\\ \sqrt{S_{CA}(i)\cdot S_{d}(i)}, & \mathrm{otherwise} \end{array}$$

where $th_{CA}$ is the adaptive threshold of the saliency map $S_{CA}$ obtained by Otsu [50] algorithm, and $S_{d}(i)=norm(d_{i})$ is the depth prior. After filtering $S_{SF}$ through the Otsu algorithm, the suppressed saliency map $S_{1}$ is obtained.

**Enhancement function**: Although the suppression function inhibits background information to a certain extent, it may lose some saliency information. The enhancement function can play a complementary role. First of all, we need to determine which depth information is reliable and needs to be retained. Here we combine three saliency maps to filter out the potential depth seed set $\Omega_{D}$ with high confidence. The depth seeds are all salient in saliency maps of $S_{fg}$, $S_{bg}$ and $S_{CA}$. The enhancement function is defined as follows,

(36) $$S_{EF}(i)=\{\begin{array}{ll} S_{1}(i)+0.2\cdot S_{d}(i), & S_{d}(i)\geq 0.9,i\in \Omega_{D}\\ S_{1}(i)+0.05\cdot S_{d}(i), & S_{d}(i)<0.9,i\in \Omega_{D}\\ S_{1}(i), & \mathrm{otherwise} \end{array}$$

After the suppression–enhancement function pair, we can get the final saliency map $S_{EF}$, which is shown in Figure 8h.

## 3. Experiments and Discussion

### 3.1. Datasets

In this part, in order to effectively demonstrate our proposed algorithm, we evaluate the model in three most popular datasets, including NLPR [13], NJU2K [26], and STERE [9]. The NLPR dataset includes 1000 RGB-D images, where the depth maps are captured by Microsoft Kinect. The NJU2K dataset contains 1985 RGB-D images which are collected from the Internet, 3-D movies and photographs taken by stereo camera, and depth maps are estimated by the optical-flow method. The STERE dataset contains 1000 stereoscopic images with the corresponding pixel-level ground truth.

### 3.2. Evaluation Metrics

Following [51], we use the following five popular evaluation metrics to evaluate the performance of the saliency detection methods comprehensively.

MAE estimates a mean absolute error between a predicted saliency map *S* and ground-truth map *GT*, it is defined as

(37) $$\mathrm{MAE}=\frac{1}{W\cdot H}\sum_{x=1}^{W}\sum_{y=1}^{H}\left|S(x,y)-GT(x,y)\right|$$

where *H* and *W* are the height and the width of the saliency map.

PR curve is formed by a series of pairs of precision and recall scores calculated at fixed thresholds ranging from 0 to 255, which describes the model performance at different situations.

(38) $$precision=\frac{\left|S\cap GT\right|}{S}$$

(39) $$recall=\frac{\left|S\cap GT\right|}{GT}$$

F-measure is a harmonic mean of average precision and recall, which is defined as,

(40) $$F_{\beta }=\frac{(1+\beta^{2})\cdot precision\cdot recall}{\beta^{2}\cdot precison+recall}$$

We empirically set $\beta^{2}=0.3$.

S-measure [52] is used to measure the spatial structure information, which is defined as,

(41) $$S_{\alpha }=\alpha \cdot S_{0}+(1-\alpha )\cdot S_{r}$$

where α is a balance parameter between the object-aware structural similarity $S_{0}$ and region-aware structural similarity $S_{r}$, and it is set to 0.5.

E-measure [53] is to evaluate the foreground map (FM) and noise, which combines local pixel values with image-level mean values to jointly capture image-level statistics and local pixel matching information.

(42) $$E_{m}=\frac{1}{W\cdot H}\sum_{x=1}^{W}\sum_{y=1}^{H}\phi_{FM}(x,y)$$

where ϕ is an enhanced alignment matrix for the two properties of a binary map.

### 3.3. Ablation Study

**Figure 9:**
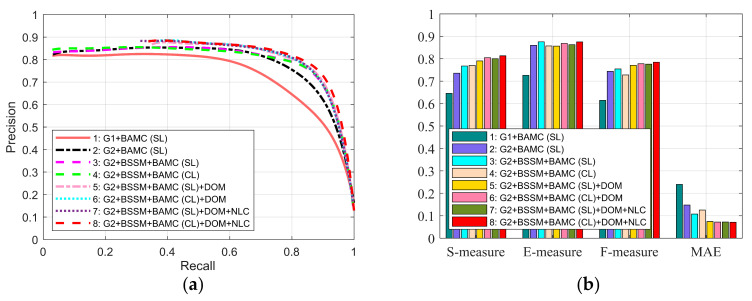
Valuation of different components. (**a**) PR curves. (**b**) S-measure, E-measure, and F-measure at adaptive threshold, mean absolute error (MAE). G1: two-layer graph; G2: two-layer spare graph; BAMC (SL): bilateral absorbing Markov chain based on single-modal multi-graph learning (color mode), CL: cross-modal multi-graph learning model (color and depth modes); BSSM: background seed screening mechanism; DOM: depth-guided optimization model; NLC: non-local connection.

Our algorithm combines background seed screening mechanism, non-local connection, cross-modal multi-graph learning model, and depth-guided optimization module. To further demonstrate the effectiveness of the components, a series of experiments are carried out. Figure 9 shows all the results of the above experiments intensively. In this part, we will combine the two-layer graph and the bilateral absorbing Markov chain based on single-modal multi-graph learning as the baseline model, which is the combination 1 in Figure 9. As is illustrated in Figure 9, the two-layer sparse graph and the background seed screening mechanism greatly improve the performance of our algorithm, which can be observed from the combinations 1, 2 and 3. Compared to the two-layer graph, the two-layer sparse graph suppresses most of the background better in Figure 5d. From Figure 5d, based on the background seed screening mechanism, background is further diluted, and the foreground is further strengthened. Compared with combination 3, the cross-modal multi-graph learning model has better improvement in precision-recall and S-measure, but the other evaluation parameters may be slightly lower. From comprehensive perspective, the cross-modal multi-image learning model and depth guided optimization module can achieve the best results which can refer to combinations 5 and 6. As Figure 6d shows, compared to the single-mode multi-graph learning model (color mode), the cross-mode multi-graph learning model can better pop foreground objects from various scenes. Figure 8h displays the effect of the depth-guided optimization module. Finally, from combinations 7 and 8, it obvious that the non-local connection can effectively improve the overall performance of the algorithm. The saliency maps with the non-local connection are more precise as shown in Figure 5g.

### 3.4. Comparisions with State-of-the-Art Methods

**Figure 10:**
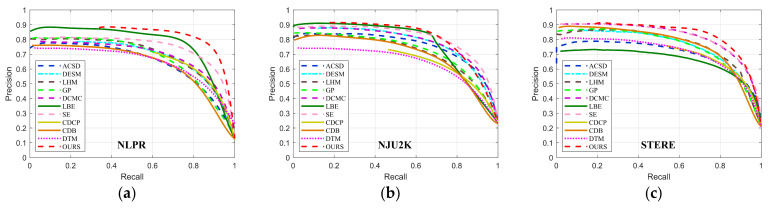
PR curves of the proposed and 10 state-of-the-art methods on 3 datasets. (**a**) NLPR dataset; (**b**) NJU2K dataset; (**c**) STERE dataset.

**Figure 11:**
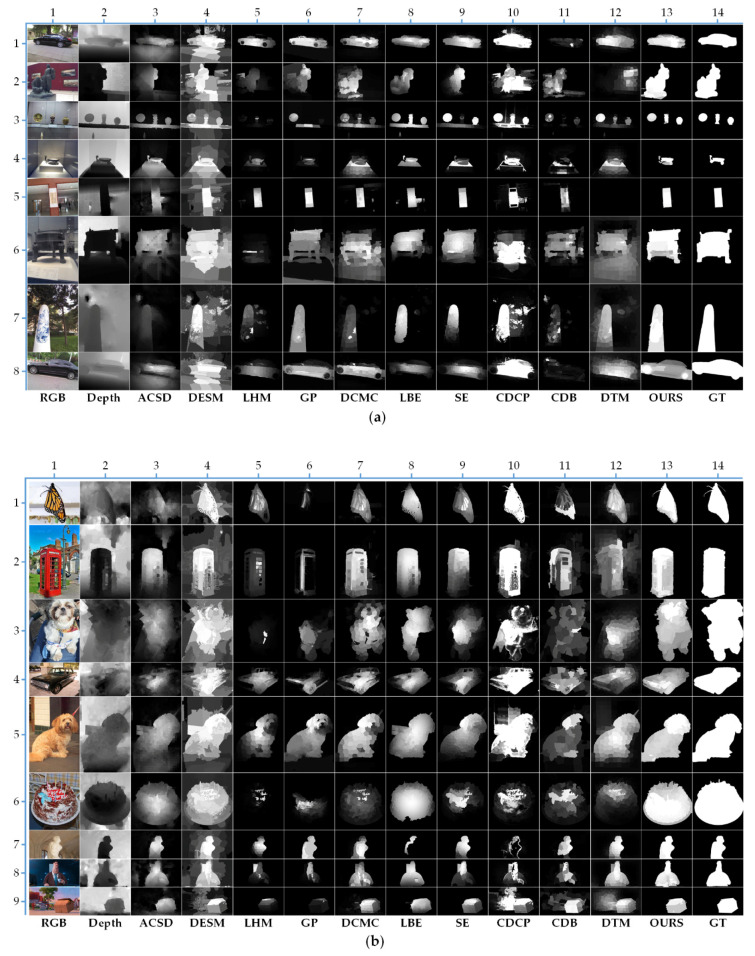

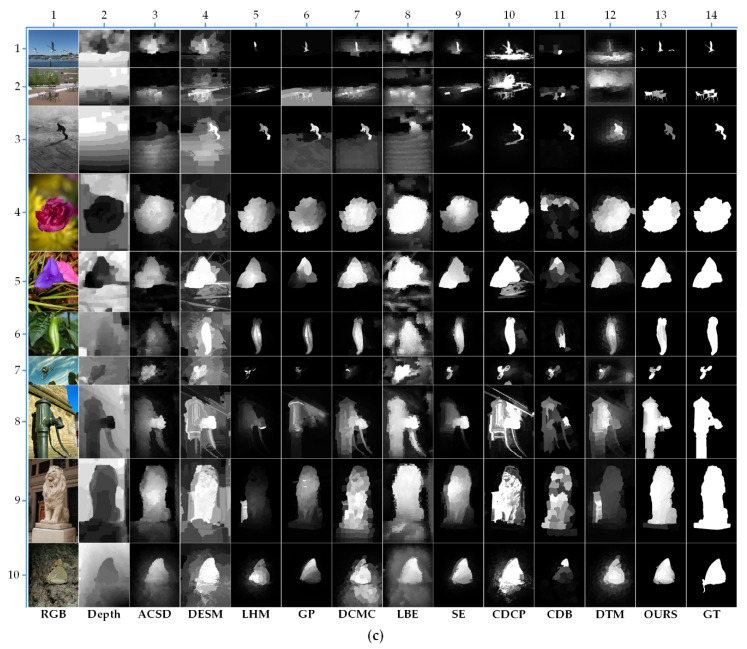
Visual examples of different methods on different datasets. (**a**) NLPR dataset; (**b**) NJU2K dataset; (**c**) STERE dataset.

We compare our proposed algorithm with 10 state-of-the-art RGB-D saliency detection models, including ACSD [26], DESM [12], LHM [13], GP [27], DCMC [37], LBE [28], SE [16], CDCP [18], CDB [24], and DTM [38]. For fair comparison, we employ saliency maps provided by the [51]. Table 1 and Figure 10 show the quantitative results of different RGBD saliency detection models. We also report saliency maps with various scenes as shown in Figure 11.

We report PR curves of three datasets in Figure 10 and list $S_{\alpha }$, $E_{m}$, $F_{\beta }$ and MAE in Table 1. As shown in Figure 10, our method achieves better PR curves on the three datasets, especially on NLPR and STERE datasets. This indicates that our method can obtain higher precision and recall compared with other methods. On the NJU2K dataset, although the end of our PR curve drops faster than some methods, we always maintain a robust curve on each dataset and keep a good balance between precision and recall overall.

As listed in Table 1, we can intuitively observe the superiority of our method among all the methods, which can be proved with the best results over all the three datasets. This demonstrates that our algorithm can generate more accurate salient regions and is more adaptable to various scenes than others.

In addition to the quantitative comparisons, to prove the effectiveness of our model visually, we also display some saliency maps in Figure 11. As we can see, the most saliency detection methods can effectively handle the cases with relatively simple backgrounds and homogenous objects. However, these methods fail to handle the complicated cases. In contrast, our method can deal with these intricate scenarios more effectively. To make it more convincing, we compare these methods in the following four aspects: (1) the effectiveness of dealing with boundary touch issues; (2) the effectiveness of the background suppression; (3) the effectiveness of solving similar appearances; and (4) the effectiveness of detection with a poor depth map.

Here combined with examples to vividly expand the above four aspects. First, as shown in the 7-th and 8-th rows of Figure 11a, the 3-th, 5-th, and 7-th rows of Figure 11b, and the 8-th row of Figure 11c, only the GP algorithm has certain resistance to boundary touch problem, but when the background is complex and the depth map is poor, as shown in the 3-th rows of Figure 11b, the detection will fail. In contrast, our algorithm achieves better results in various scenes when encountering this situation. Then, from the 3-th, 4-th, and 6-th rows of Figure 11a, we can find that most of the algorithms cannot effectively remove the background in front of the salient objects due to the interference from the depth near the camera. However, our method can availably eliminate them by using learning fusion. Moreover, as shown in the 7-th row of Figure 11b, the 8-th and 10-th rows of Figure 11c, our method works well when the color appearance of salient object is similar to the background. Finally, our model is still robust under the condition of poor depth map quality, which is demonstrated in the 3-th and 4-th rows of Figure 11b, the 1-th, 2-th, 3-th, and 6-th rows of Figure 11c.

In general, our algorithm has better robustness in the various complex scenarios. Especially, when the salient objects touch the image boundary or the depth map quality in the dataset is uneven, our method still has a good performance, which can obtain the uniform and highlighted salient objects.

**Computational complexity**. We utilize the computational complexity to prove the advantages of our proposed method compared to other methods (traditional-based and deep learning-based). In this paper, we adopt the floating point operations (FLOPs) to measure the computational complexity of the models. For fair comparisons, we obtain the deployment codes released by authors and use the same configuration as much as possible to estimate their computational complexity. As illustrated in Table 2, compared with the latest deep learning-based methods such as D^3^Net [51], BBS-Net [54], and UC-Net [55], our computational complexity is only one tenth or even one hundredth of theirs. Moreover, compared with the traditional-based methods such as DCMC [37], CDCP [18], and DTM [38], our model can achieve obvious higher performance in the relatively lower computational complexity combined with Table 1.

## 4. Conclusions and Future Work

In this paper, we propose a RGB-D saliency detection model with the bilateral absorbing Markov chain guided by depth information. Using the explicit combination of depth and color information, we first generate the low-level saliency cues based on the background prior and contrast prior. Then, to overcome the existing drawbacks in the absorbing Markov chain model, we propose a serial of methods: the background seed screening mechanism (BSSM) for boundary touch cases and the cross-modal multi-graph learning model for multi-modal fusion. Moreover, considering the limitation of local intrinsic correlation, a non-local intrinsic correlation is introduced to improved two-layer sparse graph. Based on the optimized bilateral absorbing Markov chain model, we obtain the mid-level saliency maps. Finally, we design a depth-guided optimization module to get more accurate high-level saliency map. The optimization module consists of two sub-modules: the cellular automata to optimize the integrated saliency map in the color space and the suppression-enhancement function pair to refine the saliency map in the depth space. Compared with most of the algorithms mentioned in this article, our method alleviates the boundary touch problem well and greatly suppresses the background. The comprehensive comparisons and ablation study on three RGB-D saliency detection datasets have demonstrated that the proposed method is effective and robust in various scenarios both qualitatively and quantitatively.

The literature [51] builds a new salient person (SIP) dataset with quite challenging which covers diverse real-world scenes from various viewpoints, poses, occlusion, illumination, and background. Moreover, deep learning-based RGB-D saliency detection methods [51,54,55] have developed vigorously and achieved the qualitative leap. Therefore, we look forward to extending our work to the deep learning in the future, exploring the complementarity of depth information and color information more fully, and dedicating ourselves to the studying of the saliency detection algorithm in real-world scenes.

## Figures and Tables

**Table 1:** Quantitative comparisons of different RGB-D saliency detection methods on three popular datasets. **Red**, **green** and **blue** indicate the best, second and third performances. ↑ denotes larger is better, and ↓ denotes smaller is better.

**Methods**	**Year**	NLPR				NJU2K				STERE			
		$S_{\alpha }↑$	$E_{m}↑$	$F_{\beta }↑$	MAE↓	$S_{\alpha }↑$	$E_{m}↑$	$F_{\beta }↑$	MAE↓	$S_{\alpha }↑$	$E_{m}↑$	$F_{\beta }↑$	MAE↓
**ACSD**	2014	0.6728	0.7418	0.5345	0.1787	**0.6992**	0.7863	0.6964	0.2021	0.6919	0.7932	0.6607	0.2000
**DESM**	2014	0.5722	0.6978	0.5633	0.3124	0.6648	0.6824	0.6321	0.2835	0.6425	0.6751	0.5942	0.2951
**LHM**	2014	0.6298	**0.8131**	0.6636	0.1077	0.5136	0.7082	0.6383	0.2048	0.5617	0.7700	0.7029	0.1719
**GP**	2015	0.6545	0.8045	0.6593	0.1461	0.5265	0.7161	0.6554	0.2106	0.5876	0.7842	0.7106	0.1822
**DCMC**	2016	0.7244	0.7856	0.6141	0.1167	0.6861	**0.7905**	0.7173	0.1716	**0.7306**	**0.8314**	**0.7425**	**0.1476**
**LBE**	2016	**0.7619**	**0.8550**	**0.7355**	**0.0813**	**0.6952**	**0.7913**	**0.7400**	**0.1528**	0.6601	0.7485	0.5951	0.2498
**SE**	2016	**0.7561**	0.8388	**0.6915**	**0.0913**	0.6642	0.7722	**0.7335**	**0.1687**	0.7082	**0.8250**	**0.7476**	**0.1427**
**CDCP**	2017	0.7270	0.8001	0.6076	0.1121	0.6685	0.7472	0.6238	0.1803	**0.7134**	0.7964	0.6655	0.1489
**CDB**	2018	0.6286	0.8094	0.6132	0.1142	0.6239	0.7448	0.6484	0.2028	0.6151	0.8079	0.7127	0.1655
**DTM**	2020	0.6787	0.7656	0.5271	0.1611	0.6490	0.7454	0.6082	0.2217	0.7049	0.7978	0.6585	0.1910
**OURS**	2020	**0.8131**	**0.8751**	**0.7845**	**0.0712**	**0.7361**	**0.7925**	**0.7494**	**0.1359**	**0.7774**	**0.8347**	**0.7724**	**0.1110**

**Table 2:** Computational complexity comparison with traditional-based and deep learning-based RGB-D saliency detection methods. **Red**, **green** and **blue** indicate the best, second and third performances.

**Methods**	**DCMC**	CDCP	DTM	D^3^Net	BBS-Net	UC-Net	OURS
**Year**	2016	2017	2020	2020	2020	2020	2020
**Platform**	Matlab	Matlab	Matlab	PyTorch	PyTorch	PyTorch	Matlab
**Image size**	640 × 480	640 × 480	640 × 480	224 × 224	352 × 352	352 × 352	640 × 480
**FLOPs(G)**	3.0891	**1.2565**	**0.4104**	55.0722	31.1396	16.1502	**0.2002**

## Data Availability

Data sharing not applicable.

## References

[1] Liu Z., Shi R., Shen L., Xue Y., Ngan K.N., Zhang Z. (2012). Unsupervised Salient Object Segmentation Based on Kernel Density Estimation and Two-Phase Graph Cut. IEEE Trans. Multimed..

[2] Achanta R., Susstrunk S. Saliency Detection for Content-Aware Image Resizing. Proceedings of the 2009 16th IEEE International Conference on Image Processing (ICIP).

[3] Li C., Guo J., Cong R., Pang Y., Wang B. (2016). Underwater Image Enhancement by Dehazing With Minimum Information Loss and Histogram Distribution Prior. IEEE Trans. Image Process..

[4] Jiang Q., Shao F., Gao W., Chen Z., Jiang G., Ho Y.-S. (2019). Unified No-Reference Quality Assessment of Singly and Multiply Distorted Stereoscopic Images. IEEE Trans. Image Process..

[5] Zhang H., Cao X., Wang R. Audio Visual Attribute Discovery for Fine-Grained Object Recognition. Proceedings of the AAAI Conference on Artificial Intelligence.

[6] Citak E., Bilgin G. Visual Saliency Aided SAR and Optical Image Matching. Proceedings of the 2019 Innovations in Intelligent Systems and Applications Conference (ASYU).

[7] Muthu S., Tennakoon R., Rathnayake T., Hoseinnezhad R., Suter D., Bab-Hadiashar A. (2020). Motion Segmentation of RGB-D Sequences: Combining Semantic and Motion Information Using Statistical Inference. IEEE Trans. Image Process..

[8] Patruno C., Marani R., Cicirelli G., Stella E., D’Orazio T. (2019). People re-identification using skeleton standard posture and color descriptors from RGB-D data. Pattern Recognit..

[9] Niu Y., Geng Y., Li X., Liu F. Leveraging stereopsis for saliency analysis. Proceedings of the 2012 IEEE Conference on Computer Vision and Pattern Recognition.

[10] Ciptadi A., Hermans T., Rehg J. (2013). An In Depth View of Saliency. Proceedings of the British Machine Vision Conference 2013, Bristol, UK, 9–13 September 2013.

[11] Desingh K., Madhava K.K., Rajan D., Jawahar C.V. (2013). Depth really Matters: Improving Visual Salient Region Detection with Depth. Proceedings of the British Machine Vision Conference 2013, Bristol, UK, 9–13 September 2013.

[12] Cheng Y., Fu H., Wei X., Xiao J., Cao X. Depth Enhanced Saliency Detection Method. Proceedings of the 2014 International Conference on Internet Multimedia Computing and Service.

[13] Peng H., Li B., Xiong W., Hu W., Ji R. (2014). Rgbd salient object detection: A benchmark and algorithms. Computer Vision—ECCV, Proceedings of the 2004 European Conference on Computer Vision, Prague, Czech Republic, 11–14 May 2004.

[14] Fan X., Liu Z., Sun G. Salient region detection for stereoscopic images. Proceedings of the 2014 19th International Conference on Digital Signal Processing.

[15] Sheng H., Liu X., Zhang S. Saliency analysis based on depth contrast increased. Proceedings of the 2016 IEEE International Conference on Acoustics, Speech and Signal Processing (ICASSP).

[16] Quo J., Ren T., Bei J. Salient object detection for RGB-D image via saliency evolution. Proceedings of the 2016 IEEE International Conference on Multimedia and Expo (ICME).

[17] Zhu C., Li G. A Three-Pathway Psychobiological Framework of Salient Object Detection Using Stereoscopic Technology. Proceedings of the 2017 IEEE International Conference on Computer Vision Workshop (ICCVW).

[18] Zhu C., Li G., Wang W., Wang R. An Innovative Salient Object Detection Using Center-Dark Channel Prior. Proceedings of the 2017 IEEE International Conference on Computer Vision Workshops (ICCVW).

[19] Qu L., He S., Zhang J., Tian J., Yang Q. (2016). RGBD Salient Object Detection via Deep Fusion. IEEE Trans. Image Process..

[20] Zhu C., Li G., Guo X., Wang W., Wang R., Felsberg M., Heyden A., Krüger N. (2017). A Multilayer Backpropagation Saliency Detection Algorithm Based on Depth Mining. Computer Analysis of Images and Patterns.

[21] Hangke S., Liu Z., Du H., Sun G., Le Meur O., Ren T. (2017). Depth-Aware Salient Object Detection and Segmentation via Multiscale Discriminative Saliency Fusion and Bootstrap Learning. IEEE Trans. Image Process..

[22] Tang C., Hou C. (2017). RGBD salient object detection by structured low-rank matrix recovery and Laplacian constraint. Trans. Tianjin Univ..

[23] Zhu C., Cai X., Huang K., Li T.H., Li G. PDNet: Prior-Model Guided Depth-Enhanced Network for Salient Object Detection. Proceedings of the 2019 IEEE International Conference on Multimedia and Expo (ICME).

[24] Liang F., Duan L., Ma W., Qiao Y., Cai Z., Qing L. (2018). Stereoscopic Saliency Model using Contrast and Depth-Guided-Background Prior. Neurocomputing.

[25] Huang P., Shen C.H., Hsiao H.F. RGBD Salient Object Detection using Spatially Coherent Deep Learning Framework. Proceedings of the 2018 IEEE 23rd International Conference on Digital Signal Processing (DSP).

[26] Ju R., Ge L., Geng W., Ren T., Wu G. Depth saliency based on anisotropic center-surround difference. Proceedings of the 2014 IEEE international conference on image processing (ICIP).

[27] Ren J., Gong X., Yu L., Zhou W., Yang M.Y. Exploiting global priors for RGB-D saliency detection. Proceedings of the 2015 IEEE Conference on Computer Vision and Pattern Recognition Workshops (CVPRW).

[28] Feng D., Barnes N., You S., Mccarthy C. Local Background Enclosure for RGB-D Salient Object Detection. Proceedings of the 2016 IEEE Conference on Computer Vision and Pattern Recognition (CVPR).

[29] Du H., Liu Z., Song H., Mei L., Xu Z. (2016). Improving RGBD Saliency Detection Using Progressive Region Classification and Saliency Fusion. IEEE Access.

[30] Shigematsu R., Feng D., You S., Barnes N. Learning RGB-D Salient Object Detection using background enclosure, depth contrast, and top-down features. Proceedings of the 2017 IEEE International Conference on Computer Vision Workshops (ICCVW).

[31] Han J., Chen H., Liu N., Yan C., Li X. (2018). CNNs-Based RGB-D Saliency Detection via Cross-View Transfer and Multiview Fusion. IEEE Trans. Cybern..

[32] Cong R., Lei J., Fu H., Lin W., Huang Q., Cao X., Hou C. (2019). An Iterative Co-Saliency Framework for RGBD Images. IEEE Trans. Cybern..

[33] Wang A., Wang M. (2017). RGB-D Salient Object Detection via Minimum Barrier Distance Transform and Saliency Fusion. IEEE Signal Process. Lett..

[34] Chen H., Li Y.F., Su D. Attention-Aware Cross-Modal Cross-Level Fusion Network for RGB-D Salient Object Detection. Proceedings of the 2018 IEEE/RSJ International Conference on Intelligent Robots and Systems (IROS).

[35] Hao C., Youfu L., Dan S. (2018). Multi-modal Fusion Network with Multi-scale Multi-path and Cross-modal Interactions for RGB-D Salient Object Detection. Pattern Recognit..

[36] Chen H., Li Y. Progressively Complementarity-Aware Fusion Network for RGB-D Salient Object Detection. Proceedings of the 2018 IEEE/CVF Conference on Computer Vision and Pattern Recognition.

[37] Cong R., Lei J., Zhang C., Huang Q., Cao X., Hou C. (2016). Saliency Detection for Stereoscopic Images Based on Depth Confidence Analysis and Multiple Cues Fusion. IEEE Signal Process. Lett..

[38] Cong R., Lei J., Fu H., Hou J., Huang Q., Kwong S. (2020). Going From RGB to RGBD Saliency: A Depth-Guided Transformation Model. IEEE Trans. Cybern..

[39] Comaniciu D., Meer P. (2002). Mean shift: A robust approach toward feature space analysis. IEEE Trans. Pattern Anal. Mach. Intell..

[40] Zhou L., Yang Z., Zhou Z., Hu D. (2017). Salient Region Detection Using Diffusion Process on a Two-Layer Sparse Graph. IEEE Trans. Image Process..

[41] Long J., Shelhamer E., Darrell T. Fully convolutional networks for semantic segmentation. Proceedings of the 2015 IEEE Conference on Computer Vision and Pattern Recognition (CVPR).

[42] Zhu W., Liang S., Wei Y., Sun J. Saliency optimization from robust background detection. Proceedings of the 2014 IEEE Conference on Computer Vision and Pattern Recognition.

[43] Cheng M.-M., Mitra N.J., Huang X., Torr P.H., Hu S.-M. (2015). Global contrast based salient region detection. IEEE Trans. Pattern Anal. Mach. Intell..

[44] Zhang L., Ai J., Jiang B., Lu H., Li X. (2018). Saliency Detection via Absorbing Markov Chain With Learnt Transition Probability. IEEE Trans. Image Process..

[45] Jiang B., Zhang L., Lu H., Yang C., Yang M.H. Saliency Detection via Absorbing Markov Chain. Proceedings of the 2013 IEEE International Conference on Computer Vision.

[46] Sun J., Lu H., Liu X. (2015). Saliency Region Detection Based on Markov Absorption Probabilities. IEEE Trans. Image Process..

[47] Luo H., Han G., Liu P., Wu Y. (2018). Salient Region Detection Using Diffusion Process with Nonlocal Connections. Appl. Sci..

[48] Bai S., Sun S., Bai X., Zhang Z., Tian Q. (2016). Smooth Neighborhood Structure Mining on Multiple Affinity Graphs with Applications to Context-Sensitive Similarity. Computer Vision–ECCV 2016, Proceedings of the European Conference on Computer Vision, Amsterdam, The Netherlands, 8–16 October 2016.

[49] Qin Y., Lu H., Xu Y., Wang H. Saliency detection via Cellular Automata. Proceedings of the 2015 IEEE Conference on Computer Vision and Pattern Recognition (CVPR).

[50] Nobuyuki O. (1979). A Threshold Selection Method from Gray-Level Histograms. IEEE Trans. Syst. Man Cybern..

[51] Fan D.P., Lin Z., Zhao J.X., Liu Y., Zhang Z., Hou Q., Zhu M., Cheng M.M.J.A. (2020). Rethinking RGB-D Salient Object Detection: Models, Data Sets, and Large-Scale Benchmarks. IEEE Trans. Neural Netw. Learn. Syst..

[52] Fan D., Cheng M., Liu Y., Li T., Borji A. Structure-Measure: A New Way to Evaluate Foreground Maps. Proceedings of the 2017 IEEE International Conference on Computer Vision (ICCV).

[53] Fan D., Gong C., Cao Y., Ren B., Cheng M., Borji A. (2018). Enhanced-alignment Measure for Binary Foreground Map Evaluation. arXiv.

[54] Fan D.-P., Zhai Y., Borji A., Yang J., Shao L. (2020). BBS-Net: RGB-D Salient Object Detection with a Bifurcated Backbone Strategy Network. Computer Vision–ECCV 2020, Proceedings of the European Conference on Computer Vision 2020, Glasgow, UK, 23–28 August 2020.

[55] Zhang J., Fan D.-P., Dai Y., Anwar S., Saleh F., Aliakbarian S., Barnes N.J.A.P.A. UC-Net: Uncertainty Inspired RGB-D Saliency Detection via Conditional Variational Autoencoders. Proceedings of the 2020 IEEE/CVF Conference on Computer Vision and Pattern Recognition (CVPR).

